# Supplementary figures and images for: Mechanistic studies of PFKFB2 reveal a novel inhibitor of its kinase activity
Source: PLoS One. 2025 May 22;20(5):e0317167. doi: 10.1371/journal.pone.0317167 (PMC12097583; doi:10.1371/journal.pone.0317167)

**Supplemental Figure 2**


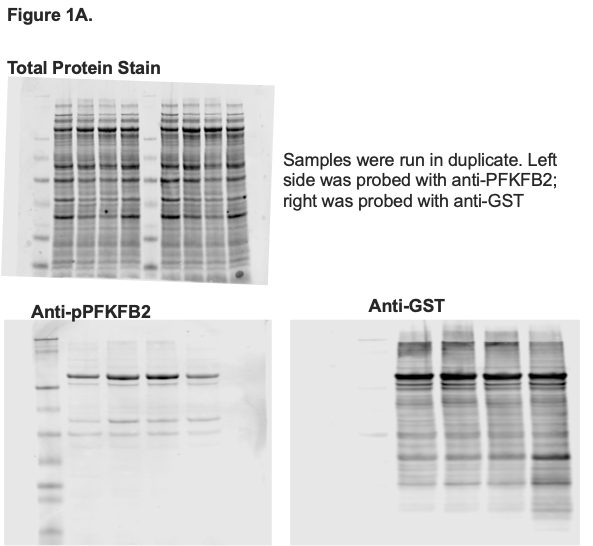


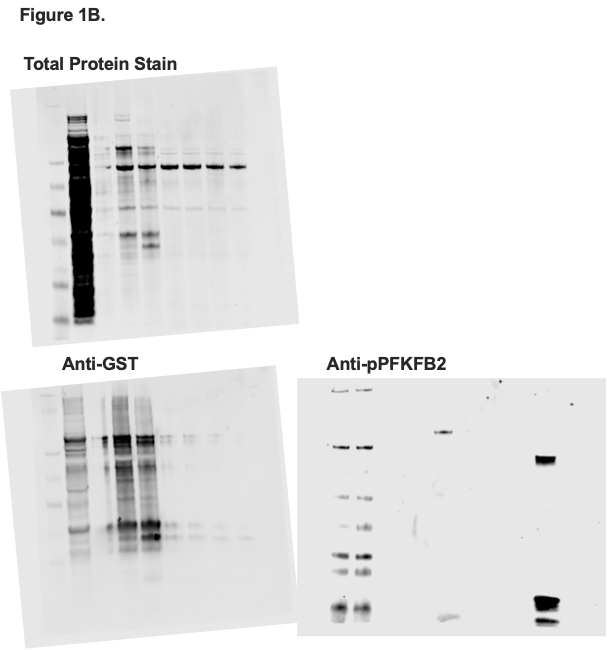


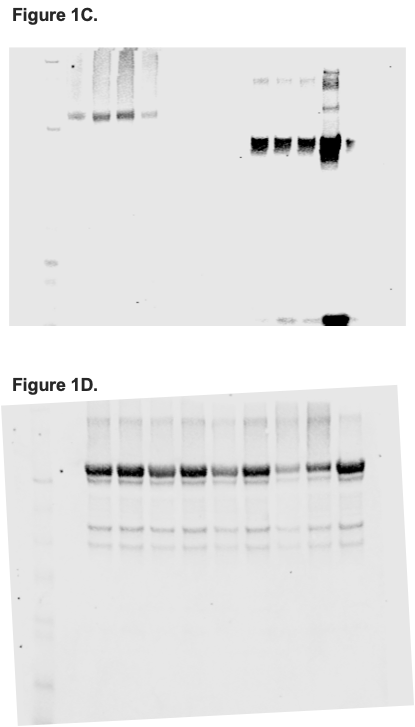

Supplement: S2 Fig — The unedited blots used in Figs 1A-D. (DOCX) [file pone.0317167.s002.docx]
